# Self-reported Cannabidiol (CBD) Use for Conditions With Proven Therapies

**DOI:** 10.1001/jamanetworkopen.2020.20977

**Published:** 2020-10-15

**Authors:** Eric C. Leas, Erik M. Hendrickson, Alicia L. Nobles, Rory Todd, Davey M. Smith, Mark Dredze, John W. Ayers

**Affiliations:** 1 The Center for Data Driven Health at the Qualcomm Institute, University of California, San Diego, La Jolla; 2 Department of Family Medicine and Public Health, University of California, San Diego, La Jolla; 3 Division of Infectious Diseases and Global Public Health, Department of Medicine, University of California, San Diego, La Jolla; 4 Department of Computer Science, Johns Hopkins University, Baltimore, Maryland

## Abstract

**Importance:**

Use of cannabidiol (CBD) has markedly increased in the past 5 years, concurrent with marketing claims that over-the-counter CBD can be used to treat almost any health condition. However, the reasons why individuals use CBD remain unclear.

**Objective:**

To assess whether individuals are using CBD for diagnosable conditions that have evidence-based therapies.

**Design, Setting, and Participants:**

This case series assessed claimed treatment applications reported by CBD users in public testimonials shared on the Reddit forum r/CBD. The r/CBD forum was selected because it includes a large, naturally occurring sample of 104 917 registered individuals who publicly discuss their experiences using CBD. All r/CBD posts were obtained from January 1, 2014, through August 31, 2019. A random sample of posts was drawn (n = 3000) and filtered to include posts in which self-identified CBD users testified why they take CBD (n = 376).

**Exposures:**

Self-reported use of CBD for medicinal purposes.

**Main Outcomes and Measures:**

Cannabidiol testimonials were divided into 11 subcategories corresponding with the condition’s medical subspecialty and 2 subcategories corresponding with wellness benefits. Posts were allowed to receive more than 1 label.

**Results:**

Of the 376 posts labeled as testimonials, 90.0% (95% CI, 86.8%-92.8%) of testimonials claimed that CBD treated the individual’s diagnosable conditions. Psychiatric conditions (eg, autism or depression) were the most frequently cited subcategory, mentioned in 63.9% (95% CI, 59.0%-69.1%) of testimonials, followed by orthopedic (26.4%; 95% CI, 21.8%-31.1%), sleep (14.6%; 95% CI, 11.3%-18.5%), and neurological (6.9%; 95% CI, 4.4%-9.6%) conditions. Testimonials also claimed that CBD treated gastroenterological conditions (3.9%; 95% CI, 1.9%-6.1%), as well as addiction, cardiological, dermatological, ophthalmological, oral health, and sexual health conditions (<2.0% each). By contrast, just 29.5% (95% CI, 24.8%-34.2%) of testimonies claimed any wellness benefit, with most citing mental wellness (eg, “quieting my mind”) (29.5% [95% CI, 24.2%-34.4%]); 1.4% (95% CI, 0.3%-2.8%) claimed a physical wellness benefit (eg, “exercise performance”).

**Conclusions and Relevance:**

The findings of this case series suggest a need for regulation of factors associated with CBD being used to treat diagnosable conditions, engagement of health care professionals with patients on their potential CBD use, and implementation of public health campaigns that encourage the public to seek treatment advice from health care professionals regarding evidence-based therapies.

## Key Points

**Question:**

Is the public using cannabidiol (CBD) to treat diagnosable conditions that have evidence-based therapies?

**Findings:**

In this case series of 376 posts on a CBD forum on Reddit, most users reported taking CBD as a therapeutic for diagnosable conditions, including mental health, cardiological, dermatological, gastroenterological, ophthalmological, oral health, and sexual health conditions, many of which have other evidence-based treatment regimens.

**Meaning:**

The findings suggest a need for interventions that address the use of CBD for unproven applications, including regulating therapeutic claims about CBD and redirecting patients to proven therapies in lieu of CBD.

## Teaser

This case series assesses whether individuals are using cannabidiol (CBD) to treat diagnosable conditions with proven therapies.

## Introduction

Since 2014, the popularity of products containing the cannabis-derived chemical compound cannabidiol (CBD) has exponentially increased in all 50 US states.^1^ This increase in popularity was concurrent with marketing claims that over-the-counter CBD can be used to treat almost any health condition, including acne, anxiety, and menstrual problems.^2^ At present, however, the only US Food and Drug Administration (FDA)–approved CBD-derived therapy is Epidiolex to treat Lennox-Gastaut syndrome and Dravet syndrome (2 rare forms of childhood epilepsy).^3^ Although other therapeutic applications of CBD have promise,^4^ at present, use of CBD to treat other conditions is not recommended by the FDA.^5^

Because of CBD marketing claims, the FDA has expressed concern by stating that “unsubstantiated therapeutic claims [are] a violation of the law [and] can put patients at risk.”^3^ However, the regulatory response of the FDA has been slow, with only a few warning letters issued^6^ and no other major actions taken. In congressional testimony, the FDA commissioner stated the agency would take stronger and wider-ranging actions if patients with diagnosable conditions were using CBD as a substitute or adjunct for approved therapies.^7^ Delayed regulatory action may in part result from the lack of available data documenting the reasons why consumers are using CBD-containing products.

To fill this knowledge gap and inform regulatory decision-making, we analyzed testimonials posted on a social media website by self-identified CBD users as part of a case series study. Although the typical methods of assessing reasons for using a product are based on surveys with active users, no surveys of CBD use among US consumers currently exist, to our knowledge. Surveys can be costly and time-consuming to field and always require some formative data to inform their need and, if necessary, their questionnaire design.^8^ Large groups of CBD users are gathering on social media to openly discuss why they use CBD, including on Reddit, a social media website with 430 million monthly users that is divided into topically focused forums termed *subreddits*.^9^ By reading these ongoing conversations, researchers can assess why the public uses CBD.^10,11,12^ Herein, we sampled posts from the subreddit r/CBD in which registered individuals publicly discuss their experiences using CBD and thematically analyzed the content of their posts to identify self-reported CBD users as well as any treatment applications they describe.

## Methods

### Study Sample

For this case series, we obtained all r/CBD posts (N = 104 917) from January 1, 2014 (the inception of r/CBD), through August 31, 2019 (the last month with publicly available data at the time of analysis). To identify use applications among CBD users, we randomly sampled 3000 original posts, with a mean of 78 words, to be further annotated. Comments were ignored because they are often nested in a stream of other comments and are typically briefer, thereby not including sufficient standalone details to be analyzed. The study was exempted from review and informed consent by the University of California, San Diego, Human Research Protections Program because the data were public and did not contain identifiable information (45 CFR §46). However, we edited direct quotations to avoid reverse identification.^13^ This study followed the reporting guideline for case series.^14^

Two of us (E.C.L. and J.W.A.) developed a codebook for identifying posts authored by individuals who were providing testimonials about their personal CBD use. A post was considered a testimonial if the user was describing their own personal use of CBD (vs an anecdote about a friend) and their intended use application (vs dosing advice). With use of the codebook, each post was labeled by 2 of 4 total coders (including E.M.H. and R.T.). Coders disagreed on 2.7% of labels and resolved disagreements through unanimous deliberation. In total, 376 posts with a mean (SD) of 174 (175) words were labeled as testimonials.

### Thematic Coding

Using open coding, 2 of us (E.C.L. and E.M.H.) reviewed the testimonials to develop a list of reasons for using CBD.^15^ There was no restriction on the number of labels for any post. Using axial coding, 4 of us (E.C.L., E.M.H., D.M.S., and J.W.A.) categorized the open codes into 2 major categories: (1) testimonials of CBD as a treatment for diagnosable conditions or (2) testimonials of CBDs for wellness. The deciding factor was whether the user reported a self- or physician-diagnosed condition with the appropriate nomenclature as opposed to citing symptoms apart from a cited diagnosis. For example, “I take CBD…to treat my bipolar disorder,” was labeled as a diagnosable condition and “CBD...makes me feel more focused” was labeled as a wellness application.

The same coders created subcategories that captured the specific intention within testimonials. Testimonials of CBD use for wellness were divided into mental and physical wellness subcategories. Testimonials of CBD use for treatment of diagnosable conditions were divided into 11 subcategories corresponding to medical subspecialties. For example, testimonials citing heart palpitations were combined into cardiological conditions. Although our thematic coding was subjective, all investigators agreed on the final definitions of the major categories (diagnosable conditions and wellness) and subcategories (Table 1).

**Table 1.** Example Posts From the Reddit Forum r/CBD

Example posts^a^	Major category		Subcategories
	Diagnosable condition	Wellness	
“Is there any legal CBD supplier that can ship to Indiana other than [BRAND NAME]? My parents were ordering the [PRODUCT] for my brother under the monthly subscription plan, but they stopped shipping to my parents. This is for relieving my brother's anxiety.”			NA
“I'm currently using [PRODUCT] but I’ve tried samples of their [PRODUCTS]. I felt like they each differed somewhat in the feeling I get. The most noticeable is that the [PRODUCT] gives me more energy. Can anyone explain the scientific differences between hemp and cannabis CBD?”		✓	Physical wellness
“My integrative physician recently suggested [PRODUCT] for addressing general anxiety disorder. My doctor recommended taking 4 drops in the morning, 5 drops in the evening, and 7 at night. Does this dosing sound correct?”	✓		Psychiatric conditions
“I've seen mixed reviews about [PRODUCT]. A local vendor that I trust and has good products started selling their stuff. After doing some research I saw that there were some bad experiences or reviews. I take CBD for ADHD and really like the clear-headedness aspect of it and also for depression. Let me know what you think of [PRODUCT] or any other [PRODUCT] you'd recommend especially if they clear your mind!”	✓	✓	Psychiatric conditions, mental wellness

Abbreviation: ADHD, attention-deficit/hyperactivity disorder; CBD, cannabidiol; NA, not applicable.

^a^ Each post was edited to omit content that might make the individual identifiable because the author may not have anticipated this content being publicly shared elsewhere and for length.

### Statistical Analysis

The monthly rates of posts to r/CBD and the percentage of testimonials with 95% CIs were calculated through bootstrapping using R, version 3.5.2 (R Project for Statistical Computing).

## Results

Posts to r/CBD increased from 1973 posts in 2016 to 6234 in 2017, 13 752 in 2018, and 11 602 during January through August 2019 (Figure). Using the random sample of r/CBD posts, we estimated that 12.1% (95% CI, 11.0%-13.4%) of all r/CBD posts were authored by individuals who provided testimonials of their use of CBD. Of the 376 posts labeled as testimonials, 90.0% (95% CI, 86.8%-92.8%) of testimonials included at least 1 claim that CBD could treat a diagnosable condition, whereas 29.5% (95% CI, 24.8%-34.2%) included at least 1 claim of a wellness benefit.

**Figure.**
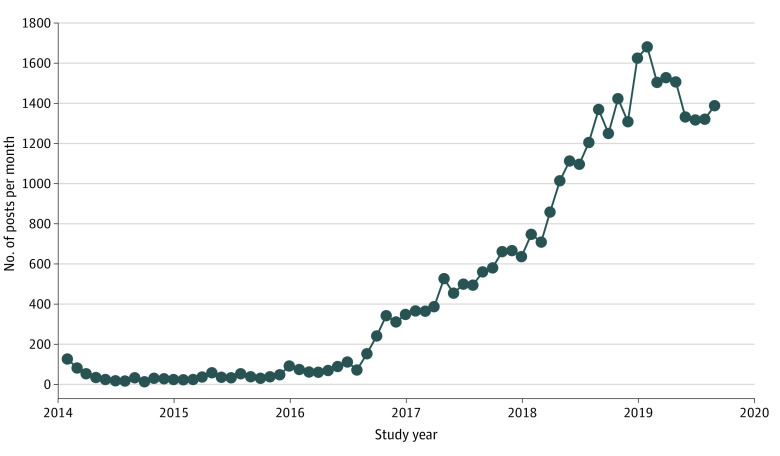
Posting Behavior on Reddit Cannabidiol Forum (r/CBD)

Psychiatric conditions were the most commonly cited diagnosable condition, mentioned in 63.9% (95% CI, 59.0%-69.1%) of testimonials (Table 2), including “after using CBD for 2 months, my autism symptoms have improved.” The second most commonly cited subcategory of diagnosable conditions was orthopedic conditions (26.4%; 95% CI, 21.8%-31.1%), followed by sleep (14.6%; 95% CI, 11.3%-18.5%), neurological (6.9%; 95% CI, 4.4%-9.6%), and gastroenterological (3.9%; 95% CI, 1.9%-6.1%) conditions. Addiction (including opioid withdrawal), cardiology (including arrhythmias), dermatology (including acne), oral health (including canker sores), ophthalmology (including vividness of color perception), and sexual health (including erectile dysfunction) were cited in fewer than 2.0% of all testimonials.

**Table 2.** Treatment Testimonials for CBD as Reported by CBD Users on Reddit

Treated condition^a^	Subcategories^a^	Relevant content from example posts^b^	Category prevalence, % (95% CI)
Diagnosable condition			
Psychiatric conditions	Attention deficit disorder, anxiety disorder, autism spectrum disorder, mood disorders (manic or depressive), borderline personality disorder, dissociation disorder, depersonalization disorder, hallucinogen persisting perception disorder, obsessive compulsive disorder, panic disorder, symptoms of psychosis, posttraumatic stress disorder, schizotypal personality disorder	“After using CBD for 2 mo, my autism symptoms have improved. My family has noticed great improvements and I have finally been able to attend important social events.”	63.9 (59.0-69.1)
Orthopedic conditions	Arthritis, ganglion cyst reduction, herniated disc, inflammation, muscle tension, muscle cramps, musculoskeletal pain, piriformis syndrome, sciatica	“I have spinal arthritis and back pain from sitting at a desk for 30 y. I like to hike but can’t do it without pain. Yesterday after taking CBD I went for a hike, it just wasn’t there at all.”	26.4 (21.8-31.1)
Sleep conditions	Insomnia, nightmares	“A life-long sufferer of insomnia, after trying CBD I finally am sleeping at night and waking up the next day feeling refreshed.”	14.6 (11.3-18.5)
Neurological conditions	Dizziness, epilepsy, headaches, Ménière disease, migraines, neurological pain, motor tics or tremors, speech disorders, vagal neuropathy	“I have been struggling with a chronic neurological illness for years that cause tremors. After trying CBD isolate, I began to feel immediate relief.”	6.9 (4.4-9.6)
Gastroenterological conditions	Appetite increase, acid reflux, gastroparesis, gastrointestinal pain, diarrhea, irritable bowel syndrome, nausea, ulcerative colitis	“I tried CBD for gastroparesis and my stomach started to get much better...after 20 y I finally feel normal.”	3.9 (1.9-6.1)
Addiction conditions	Cravings or withdrawal symptoms [including alcohol, kratom, opioids, risperidone, Δ^9^-tetrahydrocannabinol], anxiety induced by Δ^9^-tetrahydrocannabinol	“I developed an opioid addiction in the past few years, so I gave CBD a try. It’s helped me control my cravings and withdrawal symptoms as I’ve tried to get off opioids.”	1.9 (0.8-3.3)
Sexual health conditions	Erectile dysfunction, libido, menstrual disorders	“I used to suffer from erectile dysfunction, but after using CBD I can now perform.”	1.1 (0.3-2.2)
Cardiological conditions	Heart palpitations	“Other medications I used for heart palpitations gave me side effects, CBD has been much better and has helped to stop my heart palpitations.”	0.6 (0.0-1.4)
Dermatological conditions	Acne, psoriasis	“After trying CBD over a year ago, my psoriasis is much better.”	0.6 (0.0-1.4)
Oral health conditions	Aphthous ulcers, mouth sore-related pain	“I have been suffering from canker sores for many years, they historically lasted at least 10 d. After using CBD as an oral rinse, they now are only lasting 3 d.”	0.6 (0.0-1.4)
Ophthalmological conditions	Color vision deficiency	“After taking CBD I am seeing colors more vividly than I was before.”	0.3 (0.0-0.8)
Wellness			
Mental wellness	Mood control, mental acuity, stress control	“CBD has helped me to quiet my racing thoughts and mentally process things that normally overwhelmed me.”	29.5 (24.8-34.2)
Physical wellness	Enhanced energy, exercise, dietary supplement	“CBD has given me more energy for daily functions.”	1.4 (0.3-2.8)

Abbreviation: CBD, cannabidiol.

^a^ Categories were developed through open- and axial-coding processes. Further details are given in the Methods section.

^b^ Each post was edited to omit content that might make the individual identifiable because the author may not have anticipated this content being publicly shared elsewhere and for length.

Among applications of CBD for wellness, mental wellness predominated, mentioned in 29.5% (95% CI, 24.2%-34.4%) of testimonials, including “CBD has helped me to quiet my racing thoughts.” Physical wellness was cited in 1.4% (95% CI, 0.3%-2.8%) of testimonials, including “CBD has given me more energy.”

## Discussion

The public has increasingly taken to the social media platform Reddit to discuss CBD, with a increase in the rate of monthly posts that mirrors the increase and trajectory of popular interest in CBD.^1^ A large fraction of these posts were authored by CBD users who described their own experience using CBD, and most of these testimonials cited taking CBD as a therapy for a diagnosable medical condition.

Although many potential therapeutic uses of CBD remain to be explored, our findings suggest that the public already perceives CBD as an effective therapeutic for many health conditions in ways that are potentially detrimental to public health.^5^ Because CBD is not an FDA-approved treatment for nearly all the conditions cited by users who post to Reddit, CBD users may unnecessarily experience prolonged illnesses that would otherwise be alleviated with proven effective treatments. In addition, CBD use is not devoid of health risks, with known risks including liver damage and male reproductive toxic effects as well as potential drug interactions that may be associated with adverse events or diminished efficacy of approved therapies and additional unknown health risks.^5^ However, the pharmacology of CBD has not been well studied; thus, little is known about both the potential therapeutic benefits or the risks of short- or long-term use.^5^

The known and unknown risks of CBD use may be exacerbated by regulatory challenges that add context to our findings. For instance, products labeled as CBD often do not contain CBD or are mislabeled and sometimes contain psychoactive agents (eg, Δ9-tetrahydrocannabinol) not suitable for the treatments being sought by users.^16^ Moreover, CBD products may contain hazardous adulterants. For instance, nearly half of the hospitalized patients in the recent e-cigarette or vaping product use–associated lung injury outbreak vaped CBD-containing e-liquids,^17^ and other mass poisonings have been linked to synthetic cannabinoids.^18^ In these events, there is currently no track-or-trace infrastructure in which batches can be sourced and removed from the CBD supply chain. These dangers demand regulatory actions governing when, where, and how all CBD is sold, consistent with past sworn testimony by the FDA director.^7^

Clinicians could also play an important role in patient safety regarding CBD. Considering that some patients may use CBD for unexpected treatment applications (eg, heart palpitations), clinicians across specialties should inform patients that over-the-counter CBD is not an approved or recommended treatment and offer opportunities for patients to obtain efficacious treatments, as well as explain that CBD use may not be risk free. Such conversations could also provide an opportunity to identify unexplored conditions that could benefit from proven treatments.

Public health professionals should also provide accurate information. Substantial debate about erroneous health information has occurred, but few cases beyond vaccine refusal exist.^19,20^ Our findings suggest a need for accurate information about CBD. For instance, additional surveillance of CBD use that overcomes the limitations of this study seems warranted. Leaders could also begin curation campaigns, with experts participating in ongoing social media conversations and mass media campaigns providing evidenced-based CBD information.^21^

### Limitations

Although this study provides a valuable look into how the general public is using CBD, it is unclear whether the results might generalize to the larger population because no external comparisons exist and information to weight the sample of Reddit users to align with population metrics was unavailable. In general, Reddit users tend to be younger and male,^22^ but this may vary among subreddits in unknown ways. We could not document entire treatment regimens from the CBD testimonies (eg, prescribed medicines that CBD users might also be taking or replacing with CBD). Because of the small sample, types of uses of CBD that are less prevalent were potentially undocumented and changes in use over time could not be explored. Automated approaches to content analyses (eg, topic models that allow for larger samples) could potentially overcome this limitation, but were not feasible for the present study because the concepts that we describe are complex and required a multistep approach that first identified testimonials and then the condition treated and its subcategories. After more is learned about how CBD and other products are discussed in social media posts or if text is generated in a more structured format, automated analyses could uncover additional clinically relevant ways that CBD is being used by the general public. Our estimates for diagnosable conditions may be significantly undercounted because some users described symptoms that may be associated with an unrevealed diagnosis (eg, an individual with depression taking CBD for sadness apart from mentioning their diagnosis).

## Conclusions

The findings of this case series suggest that the public may already perceive CBD as an effective therapeutic for many health conditions. We believe this misperception warrants a multipronged response encompassing regulation, clinical practice, and health education. For example, regulators could enforce rules on market practices that may result in CBD being used to treat diagnosable conditions. Health care professionals could engage patients on their potential CBD use and redirect them to proven evidenced-based medicines. Public health agencies could run informational campaigns that encourage the public to seek treatment advice from health care professionals in lieu of CBD and provide vetted information on the limited proper uses of CBD for therapeutic benefit.
